# Practical tips for teaching medicine in the metaverse

**DOI:** 10.12688/mep.20445.1

**Published:** 2024-07-11

**Authors:** Miguel Angel Rodriguez-Florido, Manuel Maynar

**Affiliations:** 1 University of Las Palmas de Gran Canaria, Las Palmas de Gran Canaria, Canary Islands, Spain; 2 MOTIVA Simulation and Education based on Technology Lab, Teaching Insular, Children's and Women's Hospital, Las Palmas de Gran Canaria, Spain

**Keywords:** Undergraduate medical students, teaching medicine, metaverse, methods in medical teaching, immersive technology

## Abstract

The metaverse is based on immersive technologies such as virtual and augmented reality, body tracking, tactile sensation, etc. A growing number of studies are demonstrating the potential of the metaverse as an attractive resource for learning medicine. However, in practice, medical teachers and students often encounter significant challenges when utilizing the underlying technologies, potentially leading to frustrating learning experiences. A significant part of the teaching time is often devoted to troubleshooting technical issues with the metaverse, and the medical content itself taking a backseat until students become proficient in navigating the metaverse environment. Therefore, it is essential to fit the metaverse's underlying technologies specifically for medical education, minimizing technical hurdles for both teachers and students. In this paper, we deal with this challenge and we present a collection of practical tips that serves as a guide for medical educators making decisions in this emerging field, where they may lack prior experience. Drawing on our observation with a cohort of 776 medical students, we conclude how to effectively identify, design, or implement educational applications tailored for efficient medical learning through the metaverse. Our work may support teachers considering metaverse learning platforms for their classrooms and it is a beneficial reference for the medical education community during the initial stages of implementing the metaverse for teaching.

## Introduction

The metaverse, described as a synthetic space generated by immersive technology that envelops the users in a common interactive and connected environment, offers many opportunities for medical education ( Massetti & Chiariello, 2023; Sandrone, 2022).

 In their work, Rodriguez-Florido *et al.* (2024) show that this kind of technological environment can be efficiently used in learning medicine. Moreover, in the literature, there are several practical experiences that implement this concept under different types of immersive technology and medical topics: basic subjects ( Gloy *et al.*, 2022; Zhao *et al.*, 2020; Liimatainen *et al.*, 2021; Moro, 2023), clinical areas ( Ryan *et al.*, 2022; Werner *et al.*, 2022; Wu & Ho, 2023; Zattoni *et al.*, 2023) and even singular circumstances ( Ahmed *et al.*, 2020; Castro *et al.*, 2022; Lewis *et al.*, 2024).

However, few studies have mentioned or considered the troubles that medical teachers have when they use the metaverse in the classroom ( Elston *et al.*, 2024; Rodriguez-Florido *et al.*, 2024). They usually spend much time preparing, managing and training students in using metaverse applications instead of teaching medicine. Within a medical education context, the underlying technological platform in the metaverse should recede into the background, assuming a secondary role. The primary value of this technology lies in its ability to generate immersive scenarios that visually replicate real-world medical environments. By engaging with these simulations, students can actively participate in learning experiences that enhance their comprehension of medical concepts, perform and develop tasks that increase the understanding of a medical topic, or perceive educational aspects that, without actually carrying out the activity, are hard to acquire using traditional methods. Thus, the metaverse's application should be contingent upon its ability to enhance the learning process or provide a complementary function to existing educational resources. It should serve as a catalyst to facilitate the acquisition of medical knowledge and skills.

 Drawing on our experience in developing and utilizing technology for healthcare education, and informed by our teaching activities with 359 students across two medical subjects (Physics and Medical Technology, Otolaryngology and Medical and Surgical Stomatology and Clinical Seminars) during 2022 and 2023, we designed and implemented an educational application within the metaverse specifically tailored for medical learning. Our experimentation with these students using our application allowed us to define the key features of metaverse applications for efficient medical education. Subsequently, during the 2023/2024 academic year, our metaverse application has been adopted for teaching 417 students enrolled in five medical degree subjects (Human Anatomy II, Human Anatomy III, Otolaryngology and Medical and Surgical Stomatology, Physics and Medical Technology, and Clinical Seminars).

Thus, in this paper, and recognizing the challenges faced by medical teachers in creating effective metaverse learning scenarios, we propose some tips to guide educators in selecting, designing, validating, or collaborating on the implementation of medical education applications in the metaverse.

## Tip 1: Natural interaction through immersive technology is essential

The technology underlying the metaverse (virtual reality, augmented reality, body tracking, tactile sensation, etc.) is based on wearable devices (some types of glasses, gloves, hand controllers, etc.) that can be cumbersome and challenging for medical teachers and students. These devices frequently necessitate a significant investment of time for medical users to become accustomed to them, and may require ongoing technical support. To effectively integrate the metaverse as an educational resource in medical learning, we propose a focus on natural user interaction within metaverse applications.

By natural user interaction, we mean that the user experience within the metaverse is natural and comfortable and facilitates immersion in an artificial scenario, focusing their attention on the proposed academic content.

An illustrative example of this consideration is the use of hands within the metaverse through hand controllers. Typically, student hands are visualized as virtual representations, even though they are manipulating physical controllers to interact with the environment. This emulation can hinder a medical student's physical perception of their hands and consequently, their ability to use them effectively within the simulation. However, if the physical interface of the hand controllers seamlessly integrates with their virtual counterparts, user dexterity and manipulation skills can be significantly improved. ( Rodriguez-Florido *et al.*, 2024).

## Tip 2: The students should identify themselves

The metaverse offers an alternative to the physical space. Unfamiliarity with the underlying technologies of the metaverse can be disorienting for medical students. They might initially feel lost without their usual physical cues for movement and interaction. Consequently, it is crucial for metaverse applications to facilitate a sense of embodiment within their virtual avatars. This allows students to perform learning tasks, interact with classmates, navigate the environment, and maintain a sense of self-awareness within the virtual space.

The purpose of the metaverse in medical learning is not to replicate reality in its entirety, but rather to create a targeted emulation that aligns with specific teaching goals. This simulated environment can foster a deeper understanding of medical concepts for both educators and students.

Therefore, metaverse applications in medical education should incorporate comprehensive guides or detailed instructions to facilitate student self-identification within this alternative learning environment.

## Tip 3: The students should locate themselves

As we mentioned above, unfamiliarity with this type of learning technology is common among medical students. They are primarily interested in medical knowledge and may have limited understanding in mastering the intricacies of the metaverse interface. 

When a group of medical students are immersed in a metaverse learning activity, they are under disorientation and apprehension upon entering it. These feelings of disorientation and apprehension should be addressed to ensure that the students focus on the learning objective. They find themselves in an open space, which they know is artificial but, due to the versatility of the immersive technology, makes them feel vulnerable in the metaverse. For this reason, to leverage their sense of immersion within the metaverse they should be offered guides to help them locate themselves within the teaching scenario.

This not only benefits the flow of the class but also prevents student disorientation within the artificial space, allowing the learning activity to begin promptly.

## Tip 4: Metaverse actions should be measured

The integration of metaverse in medical education has the potential to enhance teaching methods and student learning outcomes. The intrinsic value of the metaverse in medical education lies not in the technology itself, but rather in its ability to offer a novel approach to medical instruction by virtually placing students in close proximity to patients. Furthermore, the digital nature of the metaverse uniquely facilitates the measurement of student actions within the virtual environment, providing medical teachers with valuable metrics on task performance. Therefore, we advise that metaverse applications in medical education should integrate a robust set of metrics to comprehensively assess student activity. This capability presents a valuable tool for assessing the students and their knowledge after the practical sessions in the metaverse.

## Tip 5: The students in the metaverse should collaborate with each other

Learning activities in medicine are usually performed by groups of students, promoting efficient knowledge sharing with limited resources and fostering a collaborative learning environment for students. Similarly, real-world medical practice necessitates collaboration among physicians. The immersive and interactive nature of the metaverse uniquely positions it to create a collaborative learning environment where students can work together to achieve specific educational objectives. This makes the metaverse a valuable tool for fostering teamwork and communication skills, essential for future medical professionals.

## Tip 6: The teacher should have a control dashboard of the metaverse

As in in-person classes, medical educators in the metaverse require control over the learning environment, including student identification, activity monitoring, observation of their virtual environment, guiding group learning tasks, and displaying activity results. A dedicated control dashboard, ideally located outside the metaverse, would empower the medical teacher to oversee and manage all aspects of the teaching activity, including real-time monitoring of student actions and events within the virtual environment. This functionality would significantly streamline the integration of metaverse-based learning into the daily teaching activity.

## Tip 7: The software application should have connectivity and cloud support features

Metaverse software applications often integrate connectivity features, enabling users to connect with the real world. This functionality provides access to external servers and online resources for real-time updates within the metaverse. In medical education, this connectivity is particularly valuable for enriching virtual environments with up-to-date medical information, including real-time updates to terminology and scene properties. This can significantly enhance the teaching experience and streamline collaboration between medical teachers and technical staff supporting the metaverse technology.

## Tip 8: Provide an optimal time for learning activities

As previously established, medical students lack familiarity with this type of learning environment. The duration of student exposure to the metaverse environment should be carefully considered by medical educators. Prolonged use of an immersive environment like the metaverse can lead to medical student fatigue and potentially hinder receptivity to future classroom applications. It is crucial to remember that the metaverse is a valuable supplementary tool to enhance teaching, not a replacement for existing educational methods in medicine. Effective teaching hinges on a balanced integration of diverse educational tools. The metaverse should be considered as one such tool within this pedagogical framework. Therefore, medical teachers should design immersive learning activities that optimize student engagement and achieve specific learning objectives within an appropriate timeframe within the metaverse environment.

## Tip 9: Make a balance between teaching goals and technology

The use of the metaverse as an educational tool in learning medicine should follow a teaching goal. This teaching objective should be established and prioritized by the medical teacher, not by the technical developer responsible for implementing the metaverse software application. While the medical teacher may not possess in-depth expertise in the underlying metaverse technology, they hold a profound understanding of medical education. There is a risk that the medical teacher might be overly influenced by the technical capabilities and creativity of the developer, whose focus may lie on technological innovation rather than medical education.

Therefore, medical teachers should take a leading role in guiding the development and application of metaverse technology in medical education. They must strive to achieve a balance between the established teaching goals and the technological affordances of the metaverse platform. In some cases, a simpler technological approach may be more conducive to effective learning, while in others, more advanced features may be necessary to achieve the desired educational outcomes. The role of technology should serve as a facilitator, enhancing the teaching process rather than dictating its content within medical education.

## Tip 10: Teaching goals should be balanced with realism of scenarios

The level of realism in a metaverse learning scenario should be aligned with the specific teaching objectives. While highly realistic environments can be conducive to learning, they often necessitate significant development time, substantial computing power, and extensive medical validation, which can be resource-intensive. Conversely, less visually complex scenarios may still facilitate the achievement of learning goals. Therefore, medical teachers must carefully consider the trade-offs between realism and educational efficacy when selecting or designing metaverse learning environments.

## Tip 11: Learning application should not depend on technology brand

The rapid pace of technological advancement in the metaverse landscape presents a challenge: the emergence of numerous new devices and user interfaces that allow users to experience better immersive and realistic sensations. Consequently, medical teachers must avoid becoming reliant on any singular technological platform or brand, as this could limit the future applicability and effectiveness of metaverse-based learning applications.

Our experience suggests that the most strategic approach involves developing educational applications that leverage open-source technologies. Open-source platforms benefit from the contributions of a vast developer community and are not tied to specific hardware vendors within the metaverse environment.

## Tip 12: Applications in the metaverse should prioritize user-friendliness

The underlying technologies of metaverse applications should prioritize a user-centered design, ensuring an intuitive and effortless experience for both medical teachers and students with varying levels of technical expertise. An user-friendly interface would seamlessly integrate into the existing workflow of medical education, promoting comfort and encouraging active participation from both teachers and students within the metaverse learning environment. Therefore, metaverse applications designed for medical education should prioritize user-friendliness to ensure an intuitive and effortless experience for participants.

## Conclusions

Medical education can benefit from the capacities and opportunities that the metaverse offers through immersive technologies. It may create realistic learning environments that supply limited resources, and build immersive simulations that put students right at the patient's side.

Medical teachers and students are very excited when they have a demo of an application in the metaverse for learning medicine. Nevertheless, they are usually challenged when they have to handle the immersive technologies that underlie this concept in the classroom. In general, they often spend a significant amount of time preparing for and adapting to this kind of environment when teaching medical sciences.

In this paper, we offer some practical tips for medical educators who want to leverage the metaverse in their classrooms. We propose these tips to help teachers choose, design, or even develop their own metaverse applications for medical education. In our experience, many medical teachers lack the specific training required to manage this new technological environment, and this work contributes to advising them. The metaverse strengthens medical education by bringing patients virtually closer to students within an immersive and safe learning environment.

## Data Availability

No data are associated with this article.

## References

[ref-1] Ahmed H Allaf M Elghazaly H : COVID-19 and medical education. *Lancet Infect Dis.* 2020;20(7):P777–P778. 10.1016/S1473-3099(20)30226-7 PMC7270510 32213335

[ref-2] Castro PL Maynar M Rodriguez-Florido MA : Tecnología Inmersiva para la educación en Ciencias de la Salud en situación de COVID-19. Experiencia práctica en docencia en Anatomía para el Grado de Medicina.Book Chapter. Vía Docendi - *Innovación Educativa*. Universidad de Las Palmas de Gran Canaria (Eds), 978-84-9042-445-2,2022;165–176. Reference Source

[ref-3] Elston P Canale GP Ail G : Twelve tips for teaching in virtual reality. *Med Teach.* 2024;46(4):495–499. 10.1080/0142159X.2023.2285396 38006603

[ref-4] Gloy I Weyhe P Nerenz E : Immersive anatomy atlas: learning factual medical knowledge in a Virtual Reality environment. *Anat Sci Educ.* 2022;15(2):360–368. 10.1002/ase.2095 33896115

[ref-6] Lewis KO Popov V Fatima SS : From static web to metaverse: reinventing medical education in the post-pandemic era. *Ann Med.* 2024;56(1): 2305694. 10.1080/07853890.2024.2305694 38261592 PMC10810636

[ref-7] Liimatainen K Latonen L Valkonen M : Virtual reality for 3D histology: multi-scale visualization of organs with interactive feature exploration. *BMC Cancer.* 2021;21(1): 1133. 10.1186/s12885-021-08542-9 34686173 PMC8539837

[ref-8] Massetti M Chiariello GA : The metaverse in medicine. *Eur Heart J Suppl.* 2023;25(Suppl B):B104–B107. 10.1093/eurheartjsupp/suad083 37091647 PMC10120971

[ref-9] Moro C : Utilizing the metaverse in anatomy and physiology. *Anat Sci Educ.* 2023;16(4):574–581. 10.1002/ase.2244 36545794

[ref-10] Rodriguez-Florido MA Reyes-Cabrera JJ Melián A : Feasibility of teaching and assessing medical students in the metaverse: design and features for its learning efficiency. *J New Approaches Educ Res.* 2024;13: 9. 10.1007/s44322-024-00009-6

[ref-11] Ryan G Callaghan S Rafferty A : Virtual reality in midwifery education: a mixed methods study to assess learning and understanding. *Nurse Educ Today.* 2022;119: 105573. 10.1016/j.nedt.2022.105573 36206631

[ref-12] Sandrone S : Medical education in the metaverse. *Nat Med.* 2022;28(12):2456–2457. 10.1038/s41591-022-02038-0 36385158

[ref-13] Werner H Ribeiro G Arcoverde V : The use of metaverse in fetal medicine and gynecology. *Eur J Radiol.* 2022;150: 110241. 10.1016/j.ejrad.2022.110241 35299111

[ref-14] Wu TC Ho CB : A scoping review of metaverse in emergency medicine. *Australas Emerg Care.* 2023;26(1):75–83. 10.1016/j.auec.2022.08.002 35953392

[ref-15] Zattoni F Carletti F Randazzo G : Potential applications of new headsets for Virtual and Augmented Reality in urology. *Eur Urol Focus.* 2023; S2405-4569(23)00295-X. 10.1016/j.euf.2023.12.003 38160172

[ref-5] Zhao J Xu X Jiang H : The effectiveness of virtual reality-based technology on anatomy teaching: a meta-analysis of randomized controlled studies. *BMC Med Educ.* 2020;20(1): 127. 10.1186/s12909-020-1994-z 32334594 PMC7183109

