# Supplementary material for: Practical tips for teaching medicine in the metaverse
Source: MedEdPublish (2016). 2024 Nov 12;14:54. Originally published 2024 Jul 11. [Version 3] doi: 10.12688/mep.20445.3 (PMC11530742; doi:10.12688/mep.20445.3)
Supplement: Peer review history

# Reviewer report: Reviewer response for version 3

**DOI:** 10.21956/mep.22268.r39485

**Published:** 2024-11-25

**Authors:** Fernando Salvetti, Barbara Bertagni

**Affiliations:** 1 Logosnet, Houston, TX, USA; 2 e-REAL Labs, Logosnet, New York, NY, USA; 3 Logosnet, Lugano, Tessin, Switzerland; 4 Centro Studi Logos, Turin, Italy

**Summary of the Article**

 The article offers twelve practical tips for integrating metaverse technologies into medical education, focusing on the challenges and opportunities educators face. Drawing from the authors’ experience with 776 medical students across several subjects, the paper provides actionable advice aimed at improving the teaching and learning experience in immersive virtual environments. The tips address topics such as natural interaction, collaboration, self-identification in virtual spaces, and the integration of metrics and dashboards to enhance educational outcomes. The paper emphasizes the potential of the metaverse as a supplementary teaching tool while acknowledging technical and pedagogical challenges.

 ​​​​​​ **Evaluation and Expanded Answers**

1. **Is the topic of the practical tips discussed accurately in the context of the current literature?**
  - **Answer:** Yes
  - **Comments:**
    The article effectively integrates current literature on metaverse applications in medical education. The references provided are relevant, addressing key challenges and benefits of immersive technology. However, expanding the introduction to include a broader discussion of virtual world platforms could improve the contextual depth of the article. Although the authors focus on wearable technologies, they should acknowledge alternative implementations of the metaverse, such as computer-based virtual environments, to align with the broader academic discourse.
2. **Are all factual statements correct and adequately supported by citations?**
  - **Answer:** Partly
  - **Comments:**
    While the article provides citations for many claims, some tips rely heavily on the authors' observations without adequate references to existing literature. For example:
    - **Tip 1 (Natural interaction):** The discussion of seamless hand-controller integration would benefit from references to studies on user experience in virtual environments.
    - **Tip 4 (Metrics):** Providing specific examples of validated metrics used in educational settings would strengthen the argument.
    - **Actionable Recommendation:** Incorporate additional citations to empirical studies or industry standards to substantiate these claims. This is especially critical for tips involving user interaction and performance metrics.
3. **Are arguments sufficiently supported by evidence from the published literature and/or the authors’ practice?**
  - **Answer:** Partly
  - **Comments:**
    The article is informed by the authors' extensive experience, which adds practical value. However, specific tips lack detailed evidence to support their effectiveness:
    - **Tip 3 (Locating students):** The suggestion to use visual cues such as footprints and path lines is interesting but could be supported by examples or empirical studies demonstrating their efficacy.
    - **Tip 12 (User-friendliness):** While the authors emphasize the importance of usability, there is little discussion of specific design features or interfaces that enhance user-friendliness.
    - **Actionable Recommendation:** Include data or examples from the authors' student cohort to illustrate how these tips were developed and validated. If possible, provide qualitative or quantitative results from student feedback.
4. **If evidence from practice is presented, are all the underlying source data available to ensure full reproducibility?**
  - **Answer:** Partly
  - **Comments:**
    The paper mentions the use of a "virtual dissection room" and insights from student cohorts but does not provide sufficient details about the specific technologies or methods employed. This lack of transparency limits reproducibility.
  - **Actionable Recommendation:**
    Provide additional information on:
    - The specific platforms, software, or hardware used.
    - Examples of learning activities and the metrics applied for assessment.
    - Data on student performance or feedback that informed the tips.
5. **Are the conclusions drawn balanced and justified on the basis of the presented arguments?**
  - **Answer:** Yes
  - **Comments:**
    The conclusions are well-reasoned and align with the findings presented. They emphasize the metaverse's role as a supplementary tool and acknowledge its limitations. The authors provide a balanced view, encouraging cautious adoption while advocating for teacher-led customization of virtual learning environments.

**Major Points to Address**

1. **Broader Contextualization of the Metaverse:**
  - While the paper focuses on wearable technology, it should briefly address computer-based metaverse applications (e.g., virtual worlds like Second Life) to appeal to a wider audience.
  - **Why It Matters:** This will make the article more inclusive of alternative approaches within the broader metaverse ecosystem.
2. **Citations for Key Claims:**
  - Add references to empirical studies or industry reports that support claims in Tips 1, 4, and 12.
  - **Why It Matters:** Strengthening these sections with evidence will enhance the scientific rigor of the article.
3. **Details on Metrics and Student Feedback:**
  - Provide examples of metrics used in the virtual dissection room and data from student cohorts.
  - **Why It Matters:** This will improve reproducibility and demonstrate the practical effectiveness of the proposed tips.
4. **User-Friendliness in Design:**
  - Expand Tip 12 by including specific features or design principles that promote usability in metaverse applications.
  - **Why It Matters:** Concrete examples will make this advice actionable for educators and developers.

**Minor Suggestions**

1. **Reorganize Tips for Logical Flow:**
  - For example, Tip 5 (Collaboration) could precede Tip 2 (Self-identification), as collaboration often precedes individual exploration in learning scenarios.
2. **Visual Summaries:**
  - Add a flowchart or diagram summarizing the tips to improve readability and engagement.
3. **Clarify Technological Dependencies:**
  - Emphasize that metaverse applications should not depend on proprietary technologies (Tip 11) by providing examples of successful open-source implementations.

This paper provides valuable guidance for educators exploring the use of metaverse technologies in medical education. By addressing the critiques outlined above, the authors can enhance the article’s scientific rigor and practical applicability. With these improvements, the paper has the potential to serve as an essential resource for educators and developers in this emerging field.

If evidence from practice is presented, are all the underlying source data available to ensure full reproducibility?

Partly

Is the topic of the practical tips discussed accurately in the context of the current literature

Yes

Are all factual statements correct and adequately supported by citations?

Partly

Are the conclusions drawn balanced and justified on the basis of the presented arguments?

Yes

Are arguments sufficiently supported by evidence from the published literature and/or the authors’ practice?

Partly

Reviewer Expertise:

XR and generative AI in education. Immersive and interactive learning

We confirm that we have read this submission and believe that we have an appropriate level of expertise to confirm that it is of an acceptable scientific standard, however we have significant reservations, as outlined above.

# Reviewer report: Reviewer response for version 3

**DOI:** 10.21956/mep.22268.r40014

**Published:** 2024-11-25

**Authors:** Werner Ravyse

**Affiliations:** 1 Turku University of Applied Sciences, Turku, Finland

Thank you to the authors for collating a list of tips for collaborative learning in a metaverse environment. Such writing has been long-awaited by both the scientific and teaching communities. Not just in Medical education, but higher education in general will benefit from this publication. I trust that they will continue their exploratory work and look forward to further findings they will share from it.

If evidence from practice is presented, are all the underlying source data available to ensure full reproducibility?

No

Is the topic of the practical tips discussed accurately in the context of the current literature

Yes

Are all factual statements correct and adequately supported by citations?

Partly

Are the conclusions drawn balanced and justified on the basis of the presented arguments?

Yes

Are arguments sufficiently supported by evidence from the published literature and/or the authors’ practice?

No

Reviewer Expertise:

I am an educator at a higher education institute and have twice implemented a metaverse environment in my teaching practices - I published my findings in the CDIO 2024 conference proceedings. I also work as a primary researcher in several Finnish and European-level projects that have implemented metaverse technology to a variety of use cases. I have been an author in approximately 30-40 publications concerning technology in education.

I confirm that I have read this submission and believe that I have an appropriate level of expertise to confirm that it is of an acceptable scientific standard.

# Reviewer report: Reviewer response for version 3

**DOI:** 10.21956/mep.22268.r40012

**Published:** 2024-11-21

**Authors:** Prasandhya Astagiri Yusuf

**Affiliations:** 1 Faculty of Medicine University of Indonesia, Jakarta, Indonesia; 2 Hannover Medical School, Hannover, Germany

Thank you for responding to the reviewer's comments and improving the text.

If evidence from practice is presented, are all the underlying source data available to ensure full reproducibility?

Partly

Is the topic of the practical tips discussed accurately in the context of the current literature

Yes

Are all factual statements correct and adequately supported by citations?

Yes

Are the conclusions drawn balanced and justified on the basis of the presented arguments?

Yes

Are arguments sufficiently supported by evidence from the published literature and/or the authors’ practice?

Yes

Reviewer Expertise:

Medical Technology, Health Big Data, Medical Entrepreneurship, Medical Physiology and Biophysics

I confirm that I have read this submission and believe that I have an appropriate level of expertise to confirm that it is of an acceptable scientific standard.

# Reviewer report: Reviewer response for version 3

**DOI:** 10.21956/mep.22268.r40011

**Published:** 2024-11-14

**Authors:** Ken Masters

**Affiliations:** 1 Sultan Qaboos University, Muscat, Muscat Governorate, Oman

The authors have addressed my concerns.

If evidence from practice is presented, are all the underlying source data available to ensure full reproducibility?

Not applicable

Is the topic of the practical tips discussed accurately in the context of the current literature

Yes

Are all factual statements correct and adequately supported by citations?

Yes

Are the conclusions drawn balanced and justified on the basis of the presented arguments?

Yes

Are arguments sufficiently supported by evidence from the published literature and/or the authors’ practice?

Yes

Reviewer Expertise:

Medical Informatics

I confirm that I have read this submission and believe that I have an appropriate level of expertise to confirm that it is of an acceptable scientific standard.

# Reviewer report: Reviewer response for version 2

**DOI:** 10.21956/mep.22183.r39490

**Published:** 2024-11-12

**Authors:** Werner Ravyse

**Affiliations:** 1 Turku University of Applied Sciences, Turku, Finland

The article under review intends to offer a broad high-level practical contribution to medical teachers who wish to implement metaverse technology in their teaching activities. Moreover, the article offers design guidelines for metaverse aesthetic, mechanics, and pedagogical considerations.

 While the article addresses its intentions accurately, the source of data falls into question. It is for me unclear which statements within the tips have roots in literature and which come from the claimed (n = 776) observations. The authors would strengthen their case considerably if they could provide participant utterances or examples of interaction observations that led to the forming of the presented tips - cross-referencing to existing work would further contribute to the article's credibility.

 Notwithstanding, the tips presented in this article are an accurate representation of key challenges that exist when attempting to implement metaverse technology in higher education. Although the tips can be traced to a plethora of other sources, it is beneficial for educators, researchers and metaverse designers to have them all in a single publication.

If evidence from practice is presented, are all the underlying source data available to ensure full reproducibility?

No

Is the topic of the practical tips discussed accurately in the context of the current literature

Yes

Are all factual statements correct and adequately supported by citations?

Partly

Are the conclusions drawn balanced and justified on the basis of the presented arguments?

Yes

Are arguments sufficiently supported by evidence from the published literature and/or the authors’ practice?

No

Reviewer Expertise:

I am an educator at a higher education institute and have twice implemented a metaverse environment in my teaching practices - I published my findings in the CDIO 2024 conference proceedings. I also work as a primary researcher in several Finnish and European-level projects that have implemented metaverse technology to a variety of use cases. I have been an author in approximately 30-40 publications concerning technology in education.

I confirm that I have read this submission and believe that I have an appropriate level of expertise to confirm that it is of an acceptable scientific standard, however I have significant reservations, as outlined above.

# Response

**Published:** 2024-11-14

**Authors:** Miguel Angel Rodriguez-Florido

**Affiliations:** Chair of Medical Technology at the University of Las Palmas de Gran Canaria, Spain

Dear Reviewer,

 Thank you very much for your interest in our publication and your comments.  

 Our article is the result of our practical experience in medical education for years, where we enroll approximately 150 students per academic year and subject. It offers tips to medical educators who are considering a similar initiative, informed by both relevant literature and our own research, with citations provided as appropriate.  

 Our tips may seem elementary to seasoned technical experts, but they offer substantial value to medical educators who are new to emerging technologies. Moreover, they provide a framework for approaching such technologies within the field of medical education.  

 Although your suggestions have been responded to by previous reviewers, you may find additional insights from participant utterances and interaction observations that informed our tips.

 These Spanish-language resources are available at: https://ctm.ulpgc.es/noticia/impacto-mediatico-proyecto-avrir-2022.

 We appreciate your feedback and your contribution to improve our paper.

# Reviewer report: Reviewer response for version 2

**DOI:** 10.21956/mep.22183.r39809

**Published:** 2024-11-08

**Authors:** Ken Masters

**Affiliations:** 1 Sultan Qaboos University, Muscat, Muscat Governorate, Oman

The paper addresses an important concern when using technology (in this case the metaverse) in education: acknowledging the problems and then trying to mitigate them.

 As the paper has already been reviewed, and the authors have responded and revised their manuscript appropriately, the paper has reached a polished stage, and I have no major concerns.

**I have only a few minor points on language, but these are relatively trivial:**

 •    “When a group of medical students are immersed”  “are” should be “is”.  Then the “they” in the main clause needs to change to “The students”.

 •    “are under disorientation and apprehension” should be “are disoriented and apprehensive”

 •    “a demo of an application” “demo” is colloquial, and should be avoided.

If evidence from practice is presented, are all the underlying source data available to ensure full reproducibility?

Not applicable

Is the topic of the practical tips discussed accurately in the context of the current literature

Yes

Are all factual statements correct and adequately supported by citations?

Yes

Are the conclusions drawn balanced and justified on the basis of the presented arguments?

Yes

Are arguments sufficiently supported by evidence from the published literature and/or the authors’ practice?

Yes

Reviewer Expertise:

Medical Informatics

I confirm that I have read this submission and believe that I have an appropriate level of expertise to confirm that it is of an acceptable scientific standard.

# Reviewer report: Reviewer response for version 2

**DOI:** 10.21956/mep.22183.r39442

**Published:** 2024-11-01

**Authors:** Marvin Mergen

**Affiliations:** 1 Department of Pediatric Oncology and Hematology, Saarland University, Homburg, Germany

Dear authors,

 Thanks for revising your manuscript and considering my comments. The quality has sufficiently improved.

If evidence from practice is presented, are all the underlying source data available to ensure full reproducibility?

Not applicable

Is the topic of the practical tips discussed accurately in the context of the current literature

Yes

Are all factual statements correct and adequately supported by citations?

Partly

Are the conclusions drawn balanced and justified on the basis of the presented arguments?

Yes

Are arguments sufficiently supported by evidence from the published literature and/or the authors’ practice?

Partly

Reviewer Expertise:

Virtual Reality in Medical Education

I confirm that I have read this submission and believe that I have an appropriate level of expertise to confirm that it is of an acceptable scientific standard.

# Reviewer report: Reviewer response for version 1

**DOI:** 10.21956/mep.21878.r38800

**Published:** 2024-09-16

**Authors:** Marvin Mergen

**Affiliations:** 1 Department of Pediatric Oncology and Hematology, Saarland University, Homburg, Germany

**Summary:**

 This article provides 12 tips for using metaverse technologies in medical education based on experience with 776 medical students.

**Overall review:**

 The article provides valuable statements about teaching medicine in the metaverse. Although I agree with practically all of the statements, they are very general, leaving the actual reader with the question "But how do I do that?". Reading the title, the reader would expect some more actionable points.

 Furthermore, although I see that the tips are based on your experience, the paper would benefit from some reference that support your statements.

**Abstract:** The abstract is precise and easy to follow.

**Introduction:** The introduction summarizes necessary background information while providing relevant and current references to the topic.

**Tip 1: **Based on my experience with teaching VR courses, I agree with your statement that the translation of controller action to virtual hands is confusing for some medical students. Nonetheless, it remains unclear how you imagine the "seamless" integration of the controllers' interface. Do you recommend hand tracking without controllers? Or how should a controller be designed to fit your expectations?

**Tip 2:** I support your statement. However, a reference should be mentioned here (e.g., Slater, who talks about the illusion of presence in VR)

 In addition: Which instructions should be given? I'd appreciate some examples.

**Tip 3:** Do you mean, the students should locate themselves in the real world, since they lose connection between virtual and physical space references? Please clarify.

**Tip 4:** I fully agree to use automated feedback and assessment to evaluate the students' performance. In addition, individual feedback by experts should be given.

**Tip 5:** Absolutely. XR-Technologies provide much potential for softskill training and teamwork.

**Tip 6:** I approve.

**Tip 7:** Indeed it is important to have regular online updates. Connectivity is also the basis for collaboration within the metaverse, so this should be a very basic requirement.

**Tip 8:** I agree that metaverse technologies are only a valuable addition to fill educational gaps. Inter-individual differences in familiarity with this technology make it hard to define the optimal time frame, but I agree that it shouldn't be too long to avoid fatigue and side effects.

**Tip 9: **I fully agree with this statement. Therefore it is of utmost importance that there is interdisciplinary collaboration between medical didactics experts and computer scientists when creating VR scenarios for medical education, a point we also emphasized in our recent scoping review about VR in medical education.

**Tip 10:** Yes, the necessary degree of realism is also topic of current discussions. In this point I would also emphasize the necessity of clearly defined learning goals and a rationale to use XR technologies to achieve them. 

**Tip 11:** I agree. Therefore, interdisciplinary projects within this subject should be supported by the government and the institutions to avoid dependency of private companies.

**Tip 12: **User-friendliness is the baseline that should be met before the students can fully focus on the actual educational content. This is a valuable point.

If evidence from practice is presented, are all the underlying source data available to ensure full reproducibility?

Not applicable

Is the topic of the practical tips discussed accurately in the context of the current literature

Yes

Are all factual statements correct and adequately supported by citations?

Partly

Are the conclusions drawn balanced and justified on the basis of the presented arguments?

Yes

Are arguments sufficiently supported by evidence from the published literature and/or the authors’ practice?

Partly

Reviewer Expertise:

Virtual Reality in Medical Education

I confirm that I have read this submission and believe that I have an appropriate level of expertise to confirm that it is of an acceptable scientific standard, however I have significant reservations, as outlined above.

## References

[1] : Immersion and the illusion of presence in virtual reality. *Br J Psychol* .2018;109(3) : 10.1111/bjop.12305 431-433 10.1111/bjop.12305 29781508

[2] : Reviewing the current state of virtual reality integration in medical education - a scoping review. *BMC Med Educ* .2024;24(1) : 10.1186/s12909-024-05777-5 788 10.1186/s12909-024-05777-5 39044186 PMC11267750

# Response

**Published:** 2024-09-27

**Authors:** Miguel Angel Rodriguez-Florido

**Affiliations:** Chair of Medical Technology at the University of Las Palmas de Gran Canaria, Spain

Dear Reviewer,

 Thank you very much for your contribution and comments to enhance our work.  

**Response:** We have carefully considered your suggestions and implemented them into our work. For each recommendation, we've provided actionable steps and incorporated scientific research to substantiate some of our claims.  

**Reviewer’s Comment: **Regarding virtual hand controllers or other tangible tools within the metaverse,

**Response:** ​​​​​​​we propose that all devices or instruments required by medical students for real-world interactions should be mirrored in the virtual environment. As discussed in our paper, Tip 1 aims to instill confidence in medical students as they engage in educational activities within the virtual space. Consequently, we fit the number of buttons and design hand interactions to align with optimal movements for performing educational tasks. For instance, if a student needs to grasp or release an object, a single button action suffices.  

**Reviewer’s Comment: **Concerning hand tracking on commercial devices,

**Response: **we have observed promising results, particularly for medical students unfamiliar with such technology. While we anticipate hand tracking to become the predominant interaction method within the metaverse, our experiments have revealed that many natural movements are not yet accurately tracked by existing sensors. Furthermore, for numerous tasks, medical students require clinical instruments. In these cases, emulating these instruments with physical objects (e.g., controllers) can enhance task performance and instill greater confidence in students compared to relying solely on virtual objects within the metaverse.  

**Reviewer’s Comment: **As per your feedback on Tip 2,

**Response: **we have incorporated the recommended reference (we appreciate it). In line with this suggestion, we propose that within the metaverse, each student is represented by a virtual avatar featuring a head wearing VR glasses, hands holding controllers, and feet adorned with virtual shoes. These elements should be distinguished by a unique identity color assigned to each student. By observing their virtual representation, students can readily determine their location, hand positions, and reference the floor. Moreover, this color-coding system enables instructors to easily identify individual students within the metaverse. We have included this example in the manuscript.  

**Reviewer’s Comment: **In Tip 3, we are referring to both the virtual and physical realms.

**Response: **From a physical perspective, instructors must ensure the safety of medical students as they navigate the physical environment, considering the factors discussed in this tip. Consequently, within the metaverse, medical students should have access to location indicators (e.g., virtual footprints on the floor) or movement guides (e.g., virtual path lines on the floor). These elements facilitate safe physical and virtual movement. We have included examples of these features in the paper.  

**Reviewer’s Comment: **Finally, as you suggested, Tip 7, while a fundamental requirement, is often overlooked due to its apparent simplicity. Therefore, we have decided to include it explicitly. Regarding Tip 9,

**Response: **we have incorporated your scientific paper as a reference in the manuscript.  

 We believe that these revisions have significantly enhanced the quality of the paper. Thank you.

# Response

**Published:** 2024-10-31

**Authors:** Marvin Mergen

**Affiliations:** Pediatric Oncology & Hematology, Universitat des Saarlandes - Campus Homburg, Homburg, Saarland, Germany

Dear authors, thank you for revising the manuscript, which has sufficiently improved its quality.

# Reviewer report: Reviewer response for version 1

**DOI:** 10.21956/mep.21878.r38336

**Published:** 2024-09-16

**Authors:** Prasandhya Astagiri Yusuf

**Affiliations:** 1 Faculty of Medicine University of Indonesia, Jakarta, Indonesia; 2 Hannover Medical School, Hannover, Germany

This article comprehensively provided practical tips for teaching medicine in the metaverse through the authors' experience designing and implementing metaverse app(s) for teaching 417 undergraduate medical students.

 While this article provides valuable insights, some areas require further clarification to enhance the quality and readability of the manuscript.

**Overall comments:**

 1. The sequence of tips needs to be rearranged for better flow.

 2. It is better to separate tips related to the technical and substantive aspects.

 3. To support each tip, putting references will support the evidence from practice.

**Reviewer's comments:**

 1. Introduction paragraph 3: "However, few studies have mentioned or considered the troubles that medical teachers have when they use the metaverse in the classroom (Elston et al., 2024; Rodriguez-Florido et al., 2024). They usually spend much time preparing, managing and training students in using metaverse applications instead of teaching medicine."

 The author should move this sentence to the end of the paragraph, using it as a bridge to the next one.

 To answer these questions, the author needs to add practical tips for the teachers on creating preparatory steps, educating students about using the metaverse, and effectively overcoming technical problems.

 2. Introduction paragraph 4: "Subsequently, during the 2023/2024 academic year, our metaverse application..." It's unclear what was (were) the VR application(s) used to teach 417 students. The author needs to list the application(s) to be more transparent. Different apps with different learning objectives and designs will yield different learning experiences.

 3. Tip 2: this tip is more related to VR app and module development, not the student. I suggest changing the title, such as: "Metaverse app should facilitate a sense of embodiment and incorporated with comprehensive guides"

 4. Tip 3: This is unclear since the author did not mention what kind of metaverse app the students used. Was it like a Roblox or Minecraft game with (almost) endless environment? Usually, the app is more restricted to learning specific learning objectives with a particular scenario/flow. Please clarify.

 5. Tip 4: " ... with valuable metrics on task performance." Providing examples of the metrics that can be measured in metaverse app will be more informative and engaging for the readers.

 6. Tip 5: This is better placed before Tip 2. First, highlight the metaverse collaboration and then explain how to identify and locate themselves.

 7. Tip 6: This is better placed after Tip 4 or combined with it. All the measured actions from the students in the metaverse are then summarized in the dashboard.

 8. Tip 12: The author may consider mentioning the shorter learning curve of a more user-friendly Metaverse app.

If evidence from practice is presented, are all the underlying source data available to ensure full reproducibility?

Partly

Is the topic of the practical tips discussed accurately in the context of the current literature

Yes

Are all factual statements correct and adequately supported by citations?

Yes

Are the conclusions drawn balanced and justified on the basis of the presented arguments?

Yes

Are arguments sufficiently supported by evidence from the published literature and/or the authors’ practice?

Yes

Reviewer Expertise:

Medical Technology, Health Big Data, Medical Entrepreneurship, Medical Physiology and Biophysics

I confirm that I have read this submission and believe that I have an appropriate level of expertise to confirm that it is of an acceptable scientific standard, however I have significant reservations, as outlined above.

# Response

**Published:** 2024-09-27

**Authors:** Miguel Angel Rodriguez-Florido

**Affiliations:** Chair of Medical Technology at the University of Las Palmas de Gran Canaria, Spain

Dear Reviewer, we would like to thank you for your comments and recommendations. Your suggestions will enhance our paper and its contribution to the medical teaching community.   First of all, we have reorganized the tips and incorporated additional scientific references.   Moreover, in response to your feedback:  

1. **Response: **We have reviewed the paragraph you indicated and believe that the sentence is appropriately positioned. It effectively counterbalances the preceding paragraph, which cited scientific literature supporting the advantages of the metaverse. Given that no other reviewers raised this concern, we have retained the paragraph in its original form.
  Furthermore, we have incorporated some practical actions into our recommendations to ensure their applicability in real-world scenarios.  
2. **Response: **You are correct that the student responses may vary depending on the specific application used. In our case, we have developed a resource rather than a specific application. This resource, a virtual dissection room within the metaverse, provides medical students with access to a virtual cadaver for performing dissections in the context of their respective subjects. To clarify this point, we have included additional information in the manuscript.
3. **Response: **We renamed the tip 2 as: “Metaverse applications should facilitate students identifying themselves”.   
4. **Response: **Yes, metaverse is thought of as a resource (i.e. a dissection room). We have included additional information that clarifies tip 3. Also, following your previous comment, we renamed the tip 3 as: “Metaverse applications should facilitate students locating themselves”.
5. **Response: **You are correct. We have incorporated illustrative examples of key performance metrics.  
6. **Response: **We moved tip 5 before tip 2.
7. **Response: **In the new version tip 6 is after the tip that you mentioned for the older version.  
8. **Response:** It is essential to acknowledge that all applications, including those within the metaverse, necessitate a learning curve and, consequently, a preliminary training period. However, in the context of the metaverse, and considering the observation that medical students generally exhibit limited interest in the technical intricacies of such technology, the coupling and usage times of applications may be influenced by this disparity between user experience and the profiles of students and teachers (Elston et al., 2024). Therefore, in a previous study (referenced in this paper), we advocated for the development of metaverse applications incorporating features designed to mitigate the learning curve of medical students. Consequently, tip 12 suggests that medical educators carefully consider this aspect when selecting, acquiring, or creating applications for metaverse-based teaching.

We appreciate comments and constructive suggestions of the reviewer for improving our paper.

# Reviewer report: Reviewer response for version 1

**DOI:** 10.21956/mep.21878.r38159

**Published:** 2024-08-20

**Authors:** Francisco Sendra-Portero

**Affiliations:** 1 Department of Radiology and Physical Medicine, Faculty of Medicine, Universidad de Málaga, Málaga, Spain

In this interesting article, the authors propose twelve tips to help teachers and students use Metaverse technology in medical education, based primarily on their experience with 776 students across five medical subjects over two consecutive academic years. The results from the first year have been previously published in one of the references provided.

 I agree with the authors that the Metaverse is a synthetic immersive environment that offers new opportunities for medical education, allowing the creation of interactive and connected learning spaces that can visually replicate real-world medical settings, and that this technology helps deepen medical students' understanding through engagement with realistic simulations. However, I do not agree with their concept of the Metaverse as being limited exclusively to wearable technology (some types of glasses, gloves, hand controllers, etc.). There are also computer-based Metaverse solutions that incorporate the concept of virtual worlds, which also have interesting applications in medical education. I think the authors should include virtual worlds in the concept of the Metaverse and consider computer-based technology in their tips, which would also amplify the message they intend to convey to the academic community.

 The concept of the Metaverse emphasizes sharing experiences, interacting with other humans mediated by avatars. The Metaverse is a new method of social connection, a simulated virtual space in which people can autonomously participate in social activities such as meetings, discussions, collaborations, and games, fostering unique learning opportunities. Virtual worlds (computer-keyboard-based) and Extended Reality solutions (wearable-devices-based) encounter similar challenges, such as learning curves for more natural interaction and self-identification, familiarization with technology to prevent disorientation and apprehension, collaboration, teacher oversight, and measuring actions. These are addressed in the twelve tips provided. I am sure the article would be enhanced if the introduction and abstract broadened the concept of the Metaverse to include virtual worlds technology, rather than focusing exclusively on wearable-device technology.

If evidence from practice is presented, are all the underlying source data available to ensure full reproducibility?

Not applicable

Is the topic of the practical tips discussed accurately in the context of the current literature

Yes

Are all factual statements correct and adequately supported by citations?

Partly

Are the conclusions drawn balanced and justified on the basis of the presented arguments?

Yes

Are arguments sufficiently supported by evidence from the published literature and/or the authors’ practice?

Partly

Reviewer Expertise:

Medical education through virtual worlds

I confirm that I have read this submission and believe that I have an appropriate level of expertise to confirm that it is of an acceptable scientific standard, however I have significant reservations, as outlined above.

## References

[1] : The Second Life Metaverse and Its Usefulness in Medical Education After a Quarter of a Century. *J Med Internet Res* .2024;26: 10.2196/59005 e59005 10.2196/59005 39106480 PMC11336510

# Response

**Published:** 2024-09-05

**Authors:** Miguel Angel Rodriguez-Florido

**Affiliations:** Chair of Medical Technology at the University of Las Palmas de Gran Canaria, Spain

Dear Reviewer, we are grateful for your thoughtful comments and suggestions for improving our manuscript. In a previous study, we conducted a pilot evaluation with a subset of our students to assess the feasibility and efficacy of this technology in a medical context. Building upon this foundational research, we developed educational materials tailored for specific health-related subjects that we mentioned in this work. Your observation that computer-keyboard-based solutions are synonymous with virtual environments is entirely accurate.

 Second Life, without a doubt, has been a valuable asset in educational contexts and has served as a foundational platform for many of the technology-based tools that educational community currently utilize. In fact, to illustrate the metaverse concept in our university classes, we mention the well-known game 'The Sims' as a relatable example. 

 However, in this paper and other of our research, we adopt the definition of the metaverse originally presented in the novel 'Snow Crash' (1992) by Neal Stephenson or presented in the film 'Virtuosity' (1995) by Brett Leonard. In both cases, the metaverse is depicted as an immersive environment where users can engage in physical and digital interactions within a hybrid universe. Thus, in alignment with contemporary scientific research (Massetti & Chiariello, 2023; Sandrone, 2022), cited in this work, we conceptualize the metaverse following that definition.

 Our recommendations are therefore tailored to this emerging category of immersive technologies, which are gaining traction in medical education. Moreover, from a technological perspective, the international society IEEE recently (last Aug. 29th) proposed a webinar (IEEE Metaverse Initiative and IEEE Computer Society's Involvement in Metaverse Related Standards) that aligns with this definition. In this webinar, they acknowledged the diminishing boundaries between the physical and cyber worlds and the emerging convergence of these merged realms.

 Despite the shared challenges presented by both terms in medical education, our focus in this work, due to our adoption of immersive technologies, is on providing tips based on our hands-on experience and expertise in this field. Then, in light of your comment, we believe it is more appropriate within the context of this manuscript to maintain our current definition of the metaverse. However, we will revise the manuscript to include your reference if other reviewers deem this point significant.

# Response

**Published:** 2024-09-27

**Authors:** Miguel Angel Rodriguez-Florido

**Affiliations:** Chair of Medical Technology at the University of Las Palmas de Gran Canaria, Spain

Dear Reviewer,

 We are grateful for your thoughtful comments and suggestions for improving our manuscript.   In a previous study, we conducted a pilot evaluation with a subset of our students to assess the feasibility and efficacy of this technology in a medical context. Building upon this foundational research, we developed educational materials tailored for specific health-related subjects that we mentioned in this work.  

**Response: **Your observation that computer-keyboard-based solutions are synonymous with virtual environments is entirely accurate. Second Life, without a doubt, has been a valuable asset in educational contexts and has served as a foundational platform for many of the technology-based tools that educational community currently utilize. In fact, to illustrate the metaverse concept in our university classes, we mention the well-known game 'The Sims' as a relatable example.   

 However, in this paper and other of our research, we adopt the definition of the metaverse originally presented in the novel 'Snow Crash' (1992) by Neal Stephenson or presented in the film 'Virtuosity' (1995) by Brett Leonard. In both cases, the metaverse is depicted as an immersive environment where users can engage in physical and digital interactions within a hybrid universe. Thus, in alignment with contemporary scientific research (Massetti & Chiariello, 2023; Sandrone, 2022), cited in this work, we conceptualize the metaverse following that definition. Our recommendations are therefore tailored to this emerging category of immersive technologies, which are gaining traction in medical education. Moreover, from a technological perspective, the international society IEEE recently (last Aug. 29th) proposed a webinar (IEEE Metaverse Initiative and IEEE Computer Society's Involvement in Metaverse Related Standards) that aligns with this definition. In this webinar, they acknowledged the diminishing boundaries between the physical and cyber worlds and the emerging convergence of these merged realms.  

 Despite the shared challenges presented by both terms in medical education, our focus in this work, due to our adoption of immersive technologies, is on providing tips based on our hands-on experience and expertise in this field. Then, in light of your comment, we believe it is more appropriate within the context of this manuscript to maintain our current definition of the metaverse. However, we will revise the manuscript to include your reference if other reviewers deem this point significant.   We appreciate your comments and recommendations
